# Genetics of VEGF Serum Variation in Human Isolated Populations of Cilento: Importance of VEGF Polymorphisms

**DOI:** 10.1371/journal.pone.0016982

**Published:** 2011-02-09

**Authors:** Daniela Ruggiero, Cyril Dalmasso, Teresa Nutile, Rossella Sorice, Laura Dionisi, Mario Aversano, Philippe Bröet, Anne-Louise Leutenegger, Catherine Bourgain, Marina Ciullo

**Affiliations:** 1 Institute of Genetics and Biophysics A. Buzzati-Traverso, CNR, Naples, Italy; 2 Inserm/University Paris SUD U669, Villejuif, France; 3 INSERM, U946, Paris, France; 4 University Paris Diderot, UMR-S946, Paris, France; Ohio State University Medical Center, United States of America

## Abstract

Vascular Endothelial Growth Factor (VEGF) is the main player in angiogenesis. Because of its crucial role in this process, the study of the genetic factors controlling VEGF variability may be of particular interest for many angiogenesis-associated diseases. Although some polymorphisms in the *VEGF* gene have been associated with a susceptibility to several disorders, no genome-wide search on VEGF serum levels has been reported so far. We carried out a genome-wide linkage analysis in three isolated populations and we detected a strong linkage between VEGF serum levels and the 6p21.1 *VEGF* region in all samples. A new locus on chromosome 3p26.3 significantly linked to VEGF serum levels was also detected in a combined population sample. A sequencing of the gene followed by an association study identified three common single nucleotide polymorphisms (SNPs) influencing VEGF serum levels in one population (Campora), two already reported in the literature (rs3025039, rs25648) and one new signal (rs3025020). A fourth SNP (rs41282644) was found to affect VEGF serum levels in another population (Cardile). All the identified SNPs contribute to the related population linkages (35% of the linkage explained in Campora and 15% in Cardile). Interestingly, none of the SNPs influencing VEGF serum levels in one population was found to be associated in the two other populations. These results allow us to exclude the hypothesis that the common variants located in the exons, intron-exon junctions, promoter and regulative regions of the *VEGF* gene may have a causal effect on the VEGF variation. The data support the alternative hypothesis of a multiple rare variant model, possibly consisting in distinct variants in different populations, influencing VEGF serum levels.

## Introduction

Angiogenesis, or the growth of new blood vessels, is required for any process that results in the accumulation of new tissue as well as many processes involving tissue remodelling. When the regulation of angiogenesis fails, blood vessels are formed excessively or insufficiently. It is thus a characteristic of multiple pathologies including cancer, cardiovascular disease, arthritis, psoriasis, macular degeneration, and diabetic retinopathy. In particular, insufficient angiogenesis can be a cause of ischemia, and excessive angiogenesis can result in tumor neovascularization and growth. The angiogenesis process is highly controlled through the balance of pro- and anti-angiogenic factors. VEGF is a crucial player in angiogenesis as it represents the principal pro-angiogenic factor. Throughout development, VEGF orchestrates the process of angiogenesis by regulating the growth, development, and maintenance of a healthy circulatory system[1]. During pregnancy, VEGF is involved in building the placenta. By exerting a powerful antiapoptotic action, VEGF promotes the growth of new blood vessels in tumorigenesis[2]. Because of the crucial role of VEGF, a study of the factors controlling its variability may be of particular interest for many angiogenesis-associated disease studies. The very high heritability of VEGF serum levels reported in the present study and elsewhere [3] suggests that genetic variability contributes to the variation of the trait in the population. Specific polymorphisms in the *VEGF* gene have been associated with a variation of protein levels [4], [5], [6] and with a susceptibility to several diseases, especially cancer development and progression [7]. However, no genome-wide search on this quantitative trait has been reported so far.

In this work we searched for new quantitative trait loci (QTLs) and polymorphisms influencing VEGF serum levels, in three isolated populations, each living in a different village in the remote hilly region of the Cilento and Vallo di Diano National Park, South Italy. As we recently reported [8], [9], each population is characterized by a large and unique genealogy, including the majority of the current population, the presence of inbreeding and a small number of founders.

We identified the 6p21.1 *VEGF* gene region as the main QTL for VEGF serum level variation, with a strong and consistent effect in all three populations. An additional and new QTL was detected on chromosome 3p26.3. With a weaker effect, this QTL was detected only in the combined sample of the three populations.

Focusing on the 6p21.1 signal, an extensive sequencing analysis of the *VEGF* gene was conducted in sub-samples from each of the three villages. Three SNPs were found to be significantly associated with VEGF serum levels in the village of Campora and a fourth SNP (rs41282644) was significantly associated with VEGF serum levels in the village of Cardile.

Altogether, the combination of information on linkage and association in these three population isolates with a common origin allows us to reject the hypothesis of a direct effect on VEGF serum levels of the four SNPs identified. The data suggest an effect of rarer variants, possibly different among the three populations. These results raise a crucial issue in the search for predictive and prognostic *VEGF* polymorphisms for tumors in the general population.

## Results

**Figure 1:**
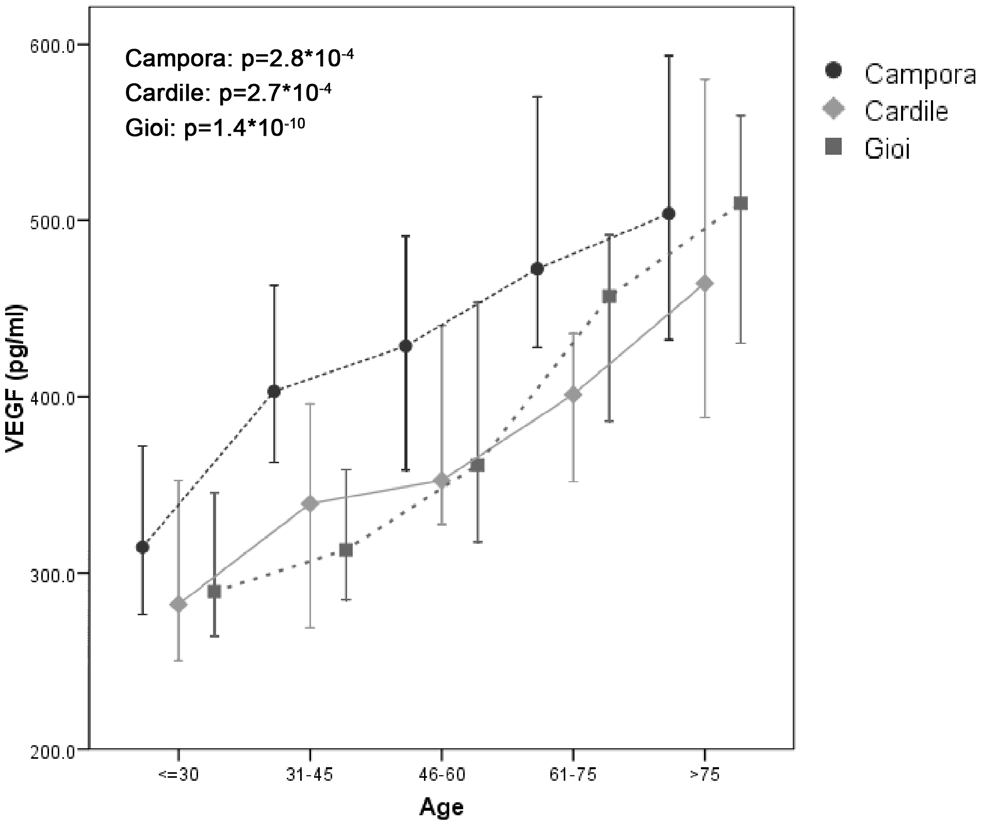
Correlation between VEGF serum levels and age in the populations of Campora, Gioi and Cardile. The increase of VEGF levels with ageing is reported in each population sample with the related p-values. In Campora, the VEGF levels are higher than in Gioi and Cardile. The median values and 95% IC of the VEGF levels for each age class are reported.

The characteristics of the study samples are reported in Table 1. The individuals of the three populations have a comparable mean age but the proportion of women is higher in Cardile. We recently reported a significant increase in VEGF serum levels with ageing in a selected sample [10]. This finding was confirmed in the complete population samples of the three villages. No difference was observed in the VEGF serum levels between men and women (Figure 1). However, the VEGF serum levels were significantly higher in Campora compared to Gioi (p-value = 3.4E-03) and Cardile (p-value = 1.4E-03), while no difference was detected between Gioi and Cardile (p-value = 0.44).

**Table 1:** Characteristics of the study samples.

Village		Campora	Gioi	Cardile
**N° of Individuals**		656	852	449
**Women %**		53.6	54.5	58.3
**Age (mean ± SE)**		49.0±0.84	49.0±0.78	48.6±0.98
**VEGF (pg/ml)**				
	**median**	413.5	374.9	355.8
**All**	**95% CI**	387.5–445.0	354.0–400.9	337.6–385.3
	**Range**	20.1–2046.6	34.3–1427.7	25.2–1589.3
	**median**	427.2	385.4	378.9
**Men**	**95% CI**	381.5–480.4	335.3–443.8	332.9–438.1
	**Range**	43.5–2046.6	42.2–1427.7	26.1–1313.3
	**median**	403.1	369.8	349.2
**Women**	**95% CI**	375.5–443.8	345.5–398.9	318.9–381.9
	**Range**	20.1–1811.7	34.3–1311.0	25.2–1589.3

### Genome-wide linkage analysis

Genome-wide linkage analysis was performed in the three population samples on the sub-pedigree sets generated by the breaking procedure applied to each population genealogy. A very strong signal was found on chromosome 6p21.1, with the highest LOD score at the marker D6S459 in Campora (mean LOD score = 7.52, q-value = 2.10E-13), in Gioi (mean LOD score = 5.31 q-value = 3.92E-04), and in the combined sample (mean LOD score = 13.94, q-value = 7.27E-22), and at the nearest marker D6S282 in Cardile (mean LOD score = 6.56, q-value = 7.01E-05) (see Table 2). The 6p21.1 region corresponds to the position of the *VEGF* gene that is exactly located at 0.5 Mb from the D6S282 marker and at 2 Mb from the D6S459 marker.

**Table 2:** Genome-wide linkage results for VEGF serum levels in the three populations and combined sample.

Sample	Chromosome	Marker	Location (cM)	Mean LOD score (min - max)*	q-value
Campora	2p16.3	D2S2156	78.42	1.98 (0.70–2.64)	0.016
	6p21.1	D6S459	72.6	7.52 (3.78–10.19)	2.10E-13
	20q13.13	D20S178	75.47	1.94 (0.86–3.47)	0.022
Gioi	6p21.1	D6S459	72.6	5.31 (1.40–7.85)	3.92E-04
Cardile	6p21.1	D6S282	68.36	6.56 (3.22–9.10)	7.01E-05
Combined sample	3p26.3	D3S4559	1.08	2.68 (0.83–4.04)	0.012
	6p21.1	D6S459	72.6	13.94 (9.15–18.99)	7.27E-22

For each sample the mean LOD scores over all sub-pedigree sets and the corresponding q-value are reported.

*value of the maximum and minimum LOD scores observed over all sub-pedigree sets.

An additional linkage was detected on chromosome 3p26.3 (mean LOD score = 2.68, q-value = 0.012) at marker D3S4559, able to reach statistical significance in the combined sample (Table 2). Additional signals were found in Campora, on chromosome 2p16.3 at marker D2S2156 (mean LOD score = 1.98, q-value = 0.016) and on chromosome 20q13.13 at marker D20S178 (mean LOD score = 1.94, q-value = 0.022) (Table 2).

### VEGF gene variability

To explore gene variability in our population, an extensive sequencing of the *VEGF* gene was carried out in a total group of 136 individuals. In detail, the exons, intron-exon junctions, promoter and regulative regions were analyzed in 42 individuals from Campora, 49 individuals from Gioi and 45 individuals from Cardile. The individuals included in these three hereafter denoted “*detection samples”* were chosen to best represent the population's genetic diversity. Data from NCBI (Assembly GRCh37) report 77 SNPs (64 SNPs and 13 Ins/Del) in the regions of the *VEGF* gene included in our analysis. In our detection samples, 36 out of the 77 (32 SNPs and 4 Ins/Del) were detected in at least one population and 18 new polymorphisms (17 SNPs and 1 Ins/Del) were identified. Two SNPs (rs3025020 and rs833070) outside the sequencing regions but available from previous studies were included in the analysis. The SNP characteristics for the three population “*detection samples”* are presented in Table 3. Note that given the “*detection sample”* sizes, all but two of the 18 new SNPs were detected in only one individual (accuracy checked with a replication of the sequencing for these rare variants, in addition to the double strand sequencing applied to all variants), the two remaining SNPs being detected in two individuals from different populations (see Table 3). A schematic representation of the position of the SNPs identified along the *VEGF* gene is reported in the supplementary figure (Figure S1).

**Table 3:** Polymorphisms identified in the *VEGF* gene through sequencing analysis of the three detection samples.

Polymorphism	Chromosome position	Gene location	Type	Minor Allele Frequency		
				Campora (N = 42)	Gioi (N = 49)	Cardile (N = 45)
new1	43735909	Promoter	A/G	A = 0.00	A = 0.01	A = 0.00
rs12208152	43735980	Promoter	C/T	T = 0.01	T = 0.01	T = 0.00
new2	43736121	Promoter	A/C	C = 0.00	C = 0.00	C = 0.03
**rs699947**	43736389	Promoter	A/C	A = 0.41	A = 0.43	A = 0.44
**rs35569394**	43736418	Promoter	Ins/Del 18 bp	Ins = 0.41	Ins = 0.44	Ins = 0.44
**rs1005230**	43736496	Promoter	C/T	T = 0.41	T = 0.44	T = 0.44
**rs35864111**	43736537	Promoter	–/G	– = 0.41	– = 0.44	– = 0.44
new3	43736625	Promoter	A/G	A = 0.00	A = 0.01	A = 0.00
**rs36208049**	43736679	Promoter	G/T	T = 0.06	T = 0.05	T = 0.09
rs36208048	43736829	Promoter	A/C	A = 0.01	A = 0.01	A = 0.00
rs36208050	43736894	Promoter	_/G	G = 0.01	G = 0.01	G = 0.00
new4	43736938	Promoter	C/T	T = 0.00	T = 0.00	T = 0.01
new5	43737384	Promoter	C/T	T = 0.00	T = 0.00	T = 0.01
**rs833061**	43737486	Promoter	C/T	T = 0.42	T = 0.50	T = 0.50
rs833062	43737529	Promoter	C/T	C = 0.02	C = 0.01	C = 0.00
rs57743727	43737698	Promoter	AG/_	– = 0.00	– = 0.00	– = 0.03
rs59260042	43737774	Promoter	A/C	A = 0.00	A = 0.01	A = 0.00
new6	43737781	Promoter	C/T	T = 0.00	T = 0.00	T = 0.02
new7	43737786	Promoter	C/T	T = 0.00	T = 0.01	T = 0.00
**rs13207351**	43737794	Promoter	A/G	G = 0.42	G = 0.50	G = 0.50
rs28357093	43737805	Promoter	A/C	C = 0.00	C = 0.00	C = 0.02
**rs1570360**	43737830	Promoter	A/G	G = 0.46	A = 0.39	A = 0.32
rs36208384	43737909	Promoter	A/C	A = 0.00	A = 0.00	A = 0.02
new8	43737983	5'UTR	C/G	C = 0.00	C = 0.02	C = 0.02
**rs2010963**	43738350	5'UTR	C/G	C = 0.43	C = 0.35	C = 0.40
**rs25648**	43738977	5'UTR	C/T	T = 0.08	T = 0.08	T = 0.20
rs56302402	43741957	intron 1	A/T	T = 0.00	T = 0.03	T = 0.03
new9	43742166	intron 2	A/C	A = 0.00	A = 0.00	A = 0.01
**rs865577**	43742419	intron 2	G/T/C	T = 0 C = 0.19	T = 0 C = 0.30	T = 0 C = 0.38
**rs833068**	43742527	intron 2	A/G	A = 0.44	A = 0.35	A = 0.44
rs833070 *	43742626	intron 2	C/T	T = 0.40	T = 0.41	T = 0.44
**rs2146323**	43745095	intron 2	A/C	A = 0.33	A = 0.19	A = 0.28
**rs3024997**	43745107	intron 2	A/G	A = 0.41	A = 0.32	A = 0.41
rs3025046	43745452	intron 3	C/G	G = 0.00	G = 0.01	G = 0.00
**rs3024998**	43745577	intron 3	C/T	T = 0.42	T = 0.34	T = 0.42
**rs3025000**	43746169	intron 3	C/T	T = 0.32	T = 0.27	T = 0.36
rs3025047	43746410	intron 4	C/T	T = 0.00	T = 0.00	T = 0.02
new10	43748302	intron 5	A/G	A = 0.01	A = 0.00	A = 0.00
rs3025015	43748350	intron 5	A/G	A = 0.00	A = 0.03	A = 0.01
**rs3025017**	43748357	intron 5	A/G	A = 0.12	A = 0.08	A = 0.08
new11	43748449	intron 5	TC/_	– = 0.00	– = 0.00	– = 0.01
**rs3025052**	43748643	intron 6	C/T	T = 0.01	T = 0.02	T = 0.07
**rs3025018**	43748795	intron 6	C/G/T	G = 0.08 T = 0.08	G = 0.05 T = 0.09	G = 0.02 T = 0.11
rs3025020 *	43749110	intron 6	C/T	T = 0.46	T = 0.26	T = 0.22
new12	43752397	3'UTR	C/T	T = 0.02	T = 0.00	T = 0.02
new13	43752518	3'UTR	C/T	T = 0.01	T = 0.00	T = 0.00
**rs3025039**	43752536	3'UTR	C/T	T = 0.14	T = 0.12	T = 0.18
new14	43752596	3'UTR	A/G	A = 0.00	A = 0.01	A = 0.00
new15	43752607	3'UTR	A/G	A = 0.00	A = 0.00	A = 0.02
new16	43753005	3'UTR	A/G	A = 0.01	A = 0.00	A = 0.00
**rs3025040**	43753051	3'UTR	C/T	T = 0.13	T = 0.11	T = 0.18
**rs10434**	43753212	3'UTR	A/G	A = 0.24	A = 0.38	G = 0.49
new17	43753292	3'UTR	C/T	T = 0.00	T = 0.00	T = 0.01
**rs3025053**	43753325	3'UTR	A/G	A = 0.07	A = 0.06	A = 0.08
**rs41282644**	43753722	3'UTR	A/G	A = 0.00	A = 0.07	A = 0.10
new18	43753882	3'UTR	A/G	G = 0.00	G = 0.01	G = 0.00

The 26 polymorphisms having a MAF>5% in at least one of the samples are reported in bold. New SNPs, not reported in the NCBI, are denoted “new”. Two SNPs (*), already available from previous studies and located outside the sequencing region, were included in the study.

### Association study on VEGF gene

In each “*detection sample”*, the SNPs with a minor allele frequency (MAF) above 5% were tested for association with the VEGF serum levels. Table 4 displays the results for all the SNPs with a significant association signal in at least one “*detection sample*” and for the SNPs repeatedly reported as associated with VEGF serum levels or correlated phenotypes in the literature. Significant associations were found between the VEGF serum levels and three common SNPs in Campora: the rs25648 variant located in the 5′UTR, the rs3025020 placed in the intron 6 and the rs3025039 located in the 3′UTR.

**Table 4:** Association results between the SNPs in the *VEGF* gene and the protein levels in the detection samples.

SNP	Campora (N = 42)			Gioi (N = 49)			Cardile (N = 45)		
	MAF	Effect (CI 95%)	p-value	MAF	Effect (CI 95%)	p-value	MAF	Effect (CI 95%)	p-value
rs699947 *	0.41	−0.19 (−0.71; 0.34)	0.486	0.43	−0.22 (−0.66; 0.23)	0.339	0.44	−0.01(−0.45; 0.43)	0.961
rs833061 *	0.42	−0.18 (−0.56; 0.20)	0.353	0.50	0.26 (−0.22; 0.74)	0.287	0.50	−0.10 (−0.62; 0.43)	0.715
rs1570360 *	G = 0.46	0.17 (−0.18; 0.53)	0.331	A = 0.39	0.46 (0.01; 0.92)	0.043	A = 0.32	−0.12 (−0.73; 0.48)	0.696
rs2010963 *	0.43	−0.06 (−0.55; 0.43)	0.799	0.35	0.04 (−0.39; 0.48)	0.840	0.40	−0.43 (−0.90; 0.04)	0.070
rs25648 *	0.08	1.34 (0.49; 2.20)	2.11E-03	0.08	0.64 (−0.04; 1.33)	0.066	0.20	−0.05 (−0.62; 0.52)	0.862
rs2146323	0.33	−0.16 (−0.70; 0.38)	0.555	0.19	−0.83 (−1.35; −0.32)	1.55E-03	0.28	−0.34 (−0.94; 0.27)	0.275
rs3025020	0.46	0.95 (0.53; 1.37)	1.01E-05	0.26	0.21 (−0.30; 0.72)	0.414	0.22	0.47 (−0.08; 1.01)	0.093
rs3025039	0.14	−1.22 (−1.93; −0.51)	7.45E-04	0.12	0.25 (−0.35; 0.85)	0.418	0.18	−0.04 (−0.68; 0.61)	0.906
rs41282644	0.00	---	---	0.07	0.31 (−0.52; 1.13)	0.463	0.10	1.27 (0.51; 2.03)	1.03E-03

The SNPs significantly associated in the *detection sample* of each population are reported. The results for the SNP (*) repeatedly associated with VEGF levels and/or related diseases in the literature are also presented.

p-value threshold corrected for multiple testing = 0.003.

These associations were confirmed in the large population sample of Campora (Table 5). The TT genotype of the rs3025039 variant was associated with lower median VEGF levels (CC = 435.9 pg/ml *vs* TT = 295.2 pg/ml) whereas the TT genotype of the rs25648 and rs3025020 variants was associated with higher levels of VEGF (CC = 382.5 pg/ml *vs* TT = 489.7 pg/ml and CC = 365.2 pg/ml *vs* TT = 447.3 pg/ml, respectively). No linkage disequilibrium was observed among these three SNPs (LD computed in the population sample: rs25648-rs3025020 r^2^ = 0.001; rs25648-rs3025039 r^2^ = 0.001; rs3025020-rs3025039 r^2^ = 0.114).

**Table 5:** Association results between the SNPs in the *VEGF* gene and the protein levels in the population samples.

SNP	Campora (N = 656)			Gioi (N = 852)			Cardile (N = 449)		
	MAF	Effect (CI 95%)	p-value	MAF	Effect (CI 95%)	p-value	MAF	Effect (CI 95%)	p-value
rs25648	0.11	0.38 (0.20; 0.55)	2.67E-05	0.09	0.13 (−0.10; 0.35)	0.276	0.11	−0.20 (−0.49; 0.10)	0.185
rs3025020	0.4	0.22 (0.10; 0.33)	3.18E-04	0.26	0.05 (−0.10; 0.20)	0.498	0.23	−0.04 (−0.27; 0.19)	0.702
rs3025039	0.17	−0.25 (−0.40; −0.10)	1.30E-03	0.15	−0.06 (−0.25; 0.12)	0.496	0.19	0.16 (−0.09; 0.41)	0.2
rs41282644	0.06	0.13 (−0.12; 0.39)	0.294	0.08	−0.06 (−0.30; 0.19)	0.655	0.11	0.59 (0.28; 0.89)	1.75E-04

Only the SNPs significantly associated in the *population sample* of each village, three SNPs in Campora and one in Cardile, are reported.

p-value threshold corrected for multiple testing = 0.01.

Surprisingly, no significant associations were found between these three SNPs and the VEGF levels in the two population samples from Gioi and Cardile (Table 5). However, the allele frequencies are not significantly different in the three populations for SNP rs25648 and rs3025039, and although rs3025020 is less frequent in Gioi and in Cardile, (0.26 and 0.23 respectively versus 0.46 in Campora), it remains a common SNP in these two villages.

Nonetheless, a variant located in the 3′UTR, rs41282644, was significantly associated with the VEGF serum levels in the “*detection sample*” of Cardile and the association was confirmed in the population sample of this village but not in the population sample of Campora nor in that of Gioi (Table 5). In Cardile, the AA genotype was associated with a lower level of VEGF (AA = 118.2 pg/ml *vs* GG = 391.2 pg/ml). In contrast to the rs25648, rs3025039 and rs3025020 SNPs, the rs41282644 SNP has a very low frequency in the Caucasian reference population (MAF = 1% in the pilot 1 CEU sample from the 1000 Genome Project) but has become more frequent in the Cilento villages (Cardile *population sample* MAF = 11%, Gioi *population sample* MAF = 8%, Campora *population sample* MAF = 6%). Note that SNP rs41282644 is not strongly correlated with the rs25648, rs3025020 and rs3025039 SNPs (r^2^ = 0.011, 0.057 and 0 with these three SNPs respectively in the *population sample* of Cardile).

One significant association was found in Gioi between the rs2146323 variant, located in the intron 2, and the VEGF serum levels. However, this association was observed only in the *detection sample* (Table 4) and was not confirmed in the population sample of Gioi.

Interestingly, the linkage disequilibrium (LD) among the four SNPs associated in at least one population was not significantly different across the populations, as suggested by the results of the global LD comparison test proposed by Zaykin et al [11] and applied to each pair of populations: Campora/Cardile, p-value = 0.23; Campora/Gioi p-value = 0.63; Gioi/Cardile p-value = 0.33 (Figure S1).

Haplotypes were also analyzed in the three population samples using successively the three SNPs associated in Campora and all the four associated SNPs (three associated in Campora and one associated in Cardile) for haplotype reconstruction. More frequent haplotypes were tested for association with the VEGF serum levels. The results show that when the three SNPs associated in Campora were considered, two haplotypes were found to be associated with the VEGF serum levels in Campora, but were not associated in Gioi and in Cardile (Table 6). Interestingly, of the two associated haplotypes, one (C-C-T haplotype) included all the alleles that in the single SNP testing were associated with low levels of VEGF, while the other (T-T-C haplotype) included all the alleles associated with high levels of VEGF. Further, the association of the T-T-C haplotype with the VEGF levels was stronger compared to that of the C-C-T haplotype and it remains statistically significant also after correction for multiple testing (Table 6).

**Table 6:** Haplotype association results.

A									
Haplotype	Frequency			Association test					
	Campora	Gioi	Cardile	Campora		Gioi		Cardile	
				Z	p-value^*^	Z	p-value	Z	p-value
**CTC**	0.383	0.208	0.176	1.75	0.080	−0.20	0.844	−0.16	0.871
**CCC**	0.381	0.575	0.566	−1.08	0.280	−1.03	0.304	0.79	0.430
**CCT**	0.133	0.127	0.161	−2.06	0.039	1.16	0.244	−0.31	0.758
**TCC**	0.049	0.062	0.064	0.44	0.662	−0.21	0.831	−0.80	0.424
**TTC**	0.027	0.017	0.028	2.79	0.005	0.12	0.901	−0.50	0.616
**TCT**	0.026	0.001	0.005	−0.44	0.657	-	-	-	-
**TTT**	0.001	0	0	-	-	-	-	-	-
**CTT**	0	0.010	0.001	-	-	-	-	-	-
*p-value threshold corrected for multiple testing = 0.008.									

B									
Haplotype	Frequency			Association test					
	Campora	Gioi	Cardile	Campora		Gioi		Cardile	
				Z	p-value*	Z	p-value	Z	p-value
**CTCG**	0.388	0.208	0.182	1.94	0.052	−0.20	0.846	−0.12	0.902
**CCCG**	0.341	0.534	0.458	−1.61	0.107	−0.91	0.365	1.07	0.285
**CCTG**	0.134	0.126	0.162	−1.40	0.161	1.34	0.180	−0.003	0.998
**CCCA**	0.045	0.048	0.102	0.24	0.810	0.01	0.989	−0.87	0.385
**TCCG**	0.039	0.046	0.064	0.17	0.865	0.08	0.939	−1.10	0.273
**TTCG**	0.024	0.016	0.025	2.37	0.018	0.14	0.892	−0.30	0.764
**TCTG**	0.021	0.001	0.004	−1.24	0.215	-	-	-	-
**TCCA**	0.005	0.01	0.002	-	-	-	-	-	-
**TCTA**	0.001	0	0	-	-	-	-	-	-
**TTCA**	0.001	0	0	-	-	-	-	-	-
**CTCA**	0.001	0.001	0	-	-	-	-	-	-
**CTTG**	0	0.01	0.001	-	-	-	-	-	-
**CCTA**	0	0	0.001	-	-	-	-	-	-
*p-value threshold corrected for multiple testing = 0.007.									

Associations between the rs25648, rs3025020 and rs3025039 haplotypes and the VEGF serum levels (A) and associations between the rs25648, rs3025020, rs3025039 and rs41282644 haplotypes and the VEGF serum levels (B) in the population samples of Campora, Gioi and Cardile are presented. Only the haplotypes with a frequency>1% were tested.

When all the four associated SNPs (three associated in Campora and one associated in Cardile) were used for haplotype reconstruction, only the T-T-C-G haplotype was still associated with the VEGF levels in Campora although only at the nominal level. No association was found between any of the haplotypes tested and the VEGF levels in Gioi and Cardile.

### Linkage on chromosome 6 conditional to VEGF SNP genotypes

**Figure 2:**
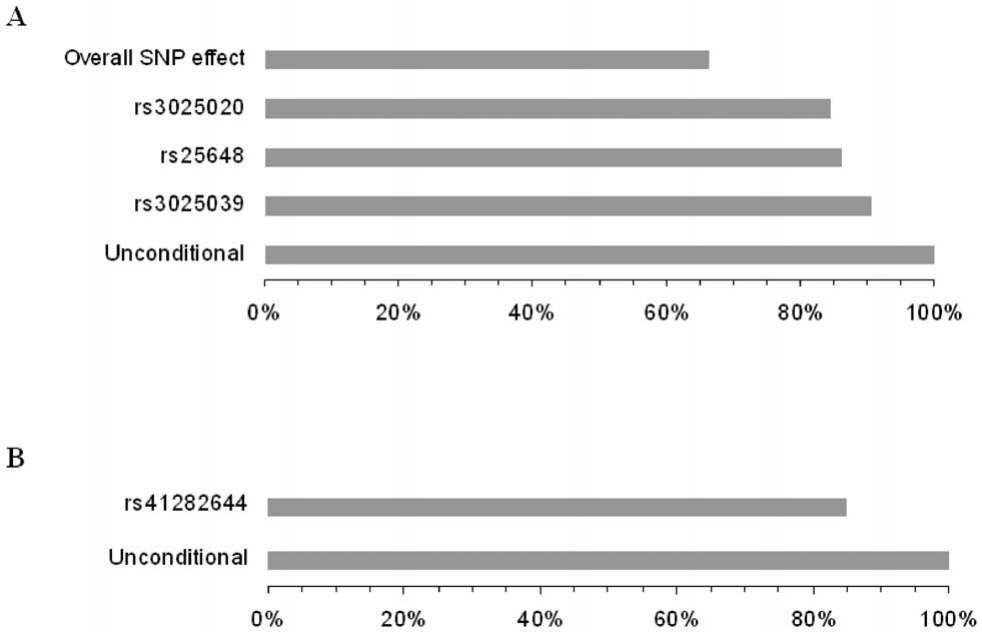
Graphical representation of the proportion of linkage explained by the SNP in Campora (A) and Cardile (B). The percentages reported correspond to the mean LOD score over all sub-pedigree sets analyzed conditional on SNP genotypes divided by the mean LOD score over all sub-pedigree sets analyzed unconditionally. A decrease of the linkage peak is observed after adjusting for the genotypes at each associated SNP. A greater effect is observed when the three SNPs detected in Campora are considered simultaneously.

To evaluate the contribution of the associated SNPs to the linkage signals detected on 6p21.1, the linkage statistics were recomputed conditional on the associated SNPs. In Campora, the original linkage peak dropped from a LOD score of 7.52 to a LOD score of 6.82 when the rs3025039 genotypes were taken into account, to a LOD score of 6.35 in the case of the rs3025020 variant and to a LOD score of 6.47 in the case of rs25648. When the linkage statistics was computed conditional on the three SNP genotypes, the LOD score dropped to LOD = 5.00, highlighting the independence of these three association signals (Figure 2). A comparable decrease of the LOD score (35%), was obtained when the linkage analysis was conditioned on each of the two haplotypes (C-C-T and T-T-C) associated with the VEGF serum levels in this population.

Similarly, the LOD score of 6.56 detected in Cardile, dropped to a LOD = 5.58 when the linkage statistics was computed conditional on the rs41282644 SNP genotypes.

The same conditional analyses were carried out in the other population samples, respectively Gioi and Cardile for SNPs rs3025039, rs3025020 and rs25648 and Gioi and Campora for SNP rs41282644. As expected, no variation in the LOD score was observed in these samples (data not shown).

## Discussion

In this study, we reported a high heritability of VEGF serum levels in our three samples (0.86, 0.80 and 0.89) and a very consistent and strong linkage of this trait with the *VEGF* gene region. Our genome-wide search detected three additional linkage signals outside the *VEGF* gene region. A signal on chr3p26 was observed but only reached significance when the three population samples were combined to increase the power, which suggests a weaker effect of this QTL. Further, no clear candidate genes could be identified in this region. Two additional signals were found on 2p16.3 and 20q13.13. Although not consistent across the populations and not detected in the combined sample, these might be of interest since interesting candidate genes are located in these regions. In fact, the EPAS1 gene, located on 2p16.3, is known to be involved in the transcriptional regulation of VEGF [12] and the NCOA-3 (SRC-3) gene, located on 20q13.13, is part of a multi-subunit co-activation complex including the p300/CBP-associated factor and the CREB binding protein [13], that participates in the induction of hypoxia-responsive genes, including the *VEGF* gene [14].

Altogether, the genome-wide linkage results suggest that most of the genetic variability accounting for the VEGF heritability comes from the *VEGF* gene region on chr6p21.

Several SNPs in the *VEGF* gene have been associated with VEGF protein levels and/or with a susceptibility to (or the severity of) several cancers such as breast, lung, colorectal, bladder prostate and gastric [6], [15], [16], [17], [18], [19]. As an increased VEGF expression has been associated with tumor progression and metastasis, these disease associations may well indirectly reflect the effect of genetic variation on VEGF levels. Among the *VEGF* SNPs, those frequently reported to be associated are: rs699947, rs833061, rs1570360, rs2010963, rs3025039 and rs25648 [7]. As recently discussed by Jain et al [7], the lack of consensus among association studies for these SNPs argues against them having a causal role in cancer development [7].

In our study, associations between rs3025039 and rs25648 and VEGF levels were detected in Campora but not in the two other villages, although these two SNPs have a similar frequency and LD pattern in all three villages. Associations with the other reported SNPs (rs699947, rs833061, rs1570360, rs2010963) could not be identified and new association signals were discovered: rs3025020 in Campora and rs41282644 in Cardile.

From the analysis of haplotypes involving the rs25648- rs3025020- rs3025039 SNPs, we note that in Campora the T-T-C haplotype is more strongly associated with the VEGF levels than the C-C-T haplotype and that it is still associated when the rs41282644 G allele was added (T-T-C-G) to the haplotype. However, the overall haplotype association results, although interesting, are less significant then the single SNP association results, as expected given that all of these are common SNPs and there is a very low LD between them.

All the associated SNPs in our study contribute to the linkage signal, but none of them explains the majority of the signal, even when considered together but independently (3 SNPs in Campora explain 35% of the linkage signal) or as a haplotype. The detection of different association signals in populations with a very similar genetic background and in which a strong linkage was detected, strongly suggests that these cannot point to functional variants, but only to proxies correlated to the functional variants.

Whether these variants are more likely to be rare or common, different or similar among populations remains an open question. Still, given that the LD patterns among common SNPs in the region are relatively similar, if common causal variants were involved, their association with rs3025039, rs25648, rs3025020 or rs41282644 should not be specific to Campora or Cardile. These SNPs should be proxies in all three populations. On the contrary, rare variants, more sensitive to genetic drift, could well display a discordant LD pattern with common variants among the three populations, explaining the discordant association results. A further study of the region, including a sequencing of the whole linkage region in larger sets of individuals, will be required to elucidate this hypothesis.

From a more methodological perspective, this work suggests that our study design, able to provide complementary information on linkage and association in three isolated populations with similar common genetic variations but possibly divergent rarer variations, is particularly powerful in a discrimination between causal and non causal variants.

## Materials and Methods

### Population sample and VEGF measurement

The study includes 1,957 individuals, recruited through a population-based sampling strategy in three small isolated villages of the Cilento region, South Italy: 656 individuals from the village of Campora, 852 from the village of Gioi and 449 from the village of Cardile. The recruited sample represents about 85% of the living population of each village. Blood samples were collected in the morning after the participants had been fasting for at least 12 h. Aliquots of serum were immediately prepared and stored at −80°C, and were subsequently used for the assessment of VEGF levels. VEGF (pg/ml) was measured using an enzyme-linked immunosorbent assay, according to the manufacturer's instructions (Quantikine™, R&D Systems, Minneapolis, MN). The study design was approved by the ethics committee of Azienda Sanitaria Locale Napoli 1. The study was conducted according to the criteria set by the declaration of Helsinki and each subject signed an informed consent before participating in the study.

Mann-Whitney U test to compare median values in independent samples was performed to compare the VEGF serum levels among population samples. Kruskal-Wallis test was applied to assess the influence of age on VEGF serum variation. These analyses were performed with the SPSS software.

### Microsatellite Genotyping

A genome-wide scan of 1,122 microsatellites (average marker spacing of 3.6 cM and mean marker heterozygosity of 0.70) was performed by the deCODE genotyping service. All subjects having a VEGF measurement were genotyped. Mendelian inheritance inconsistencies were checked with the Pedcheck program[20].

### Pedigree breaking and linkage analysis

In each village, the vast majority of the phenotyped individuals were connected through a unique deep pedigree. In Campora, 627 out of the 656 phenotyped individuals were included in a 3,049-member pedigree. In Gioi, 798 out of the 852 phenotyped individuals were related through a 4,190-member pedigree. In Cardile, a pedigree of 2,384 members connected 425 individuals out of the 449 phenotyped individuals.

The heritability of VEGF serum levels was estimated using the SOLAR software [21]. A log-transformation was applied to the trait to eliminate an excess of kurtosis. Gender and age were tested as covariates, and only age was retained in the final model. Residuals of the covariate regression were normally distributed and used for heritability estimations. The estimations of heritability were 0.86, 0.80 and 0.89 in Campora, Gioi and Cardile respectively.

The linkage analysis was performed following a procedure based on a multiple splitting of the genealogy, that we developed and already applied to various complex traits [22], [23]. This approach capitalizes on the fact that different family structures differ in their power to detect linkage [24] by successively considering the use of different splittings of the population pedigree. Different splittings of each large population genealogy into sets of sub-pedigrees were generated following a procedure that we previously described [25]. Briefly, the sub-pedigree sets were obtained with the clique-partitioning Jenti method [26], applying different constraints on the splitting procedure (minimum and maximum clique size, minimum relationship level among clique members, and maximum complexity of the resulting families). A selection of the most informative sets was made by maximizing the number of related phenotyped pairs of individuals included in the sets and by minimizing the similarity among the sets in terms of number of pairs in common. By using this approach 15 sub-pedigree sets in Campora, 16 in Gioi, 18 in Cardile and 25 in the combined sample were obtained. The characteristics of these sub-pedigree sets are reported in a supplementary table (Table S1).

A linear regression model of the log-transformed VEGF on age was applied and the residuals were used as a quantitative trait in the multipoint quantitative linkage analysis on each sub-pedigree set using the regression-based approach implemented in MERLIN-REGRESS [27]. The population mean and variance of VEGF were computed from all phenotyped individuals in each population separately, and in the combined sample for the combined analysis.

The contribution of the associated polymorphisms to the linkage signal on chromosome 6 was assessed by performing the linkage analysis on a new phenotype: the VEGF levels adjusted for age and SNP genotypes with a genotypic modeling of the SNP effect.

To take into account the multiple testing problem created by both the number of markers tested and the number of pedigree sets analyzed, we considered a parametric false discovery rate (FDR) approach.

For each marker in each population, the mean LOD score statistics over all the sub-pedigree sets was transformed into a test statistic with a theoretical null distribution following a standard normal [28]. Indeed, to estimate the q-values (which are, for each marker, the minimum FDR induced by the rejection of the null hypothesis), a modelization of the marginal distribution of the test statistic is required and the transformed test statistic is more easily modelized.

A K-components Gaussian mixture model with equal variances was chosen to modelize the marginal distribution of the transformed test statistics, as such a mixture model efficiently separates the empirical null distribution (likely to be composite and different from the theoretical one [28], [29]) from the alternative distribution. For a range of K values (from 2 to 15), the model parameters were inferred in a Bayesian framework by sampling from their joint posterior distributions using MCMC samplers implemented in the WinBUGS software [30]. From the different models, corresponding to different values of K, we selected the one having the highest log-likelihood. To estimate the q-values, without neglecting the fact that the empirical null distribution may be different from the theoretical one [28], [29], the null distribution in the mixture model was itself modelized by the mixture of the K_0_ first components (K_0_≤K). K_0_ was chosen such that the L1 distance between the estimated null density and the density of the theoretical null distribution (a standard normal distribution) was the minimum. Finally, we report here the markers with a q-value below 5%[29], [31].

### Identification and genotyping of SNPs in the *VEGF* gene

To identify polymorphisms in the *VEGF* gene, the exons, intron-exon junctions, promoter and regulative regions were sequenced in the “*detection samples”* of individuals selected to best represent the genetic diversity of each village while maintaining reasonable sample sizes (42 individuals in Campora, 45 in Cardile and 49 in Gioi). All the individuals included in the “*detection samples”* were among the oldest individuals for which DNA was available with children, grand-children and great-grandchildren included in the population sample. The mean number of direct descendants (children, grandchildren and great-grandchildren) was 5.8 for the 42 individuals included in the Campora detection sample, 8.3 for the 45 individuals included in the Cardile detection sample and 9.12 for the 49 individuals included in the Gioi detection sample.

Altogether, 9.8 kb, corresponding to 50% of the entire gene, were analyzed. The oligonucleotide primers for the amplification and sequencing of these regions were designed using the primer prediction program Primer3 (Table S2). The PCR fragments were obtained by 20 µl reaction containing 0.2 of mM dNTPs, 0.8 µM of each forward and reverse primer, 1.5 mM of MgCl_2_, and 40 ng of genomic DNA as template, with 2 units of recombinant Taq DNA polymerase. The cycling conditions were as follows: 95°C for 3 min, followed by 95°C for 30 sec, 60°C for 30 sec, and 72°C for 30 sec for 35 cycles, and by a final extension at 72°C for 7 min. The PCR products were purified by using MultiScreen PCR_µ_96 Filter Plates (Millipore) and were sequenced on both strands using the Applied Biosystems BigDye v3.1 sequencing kit according to the manufacturer's recommendations on an Applied Biosystems 3730 DNA Analyzer Sequencer. The sequences were then analyzed using the SeqAnalysis and BioEdit softwares. The SNP discovery accuracy was assured by sequencing in two replicates the fragments including the new SNPs.

As mentioned in the *Results* section, two SNPs were added to this panel: rs833070 (located in intron 2) and rs3025020 (located in intron 6) and genotyped using the TaqMan SNP genotyping assay and the SDS software was used for allele discrimination (Applied Biosystems, Foster City, CA, USA). The same technology was used to genotype in the population samples the five SNPs associated in the “*detection samples”* (rs25648, rs3025039, rs3025020, rs41282644 and rs2146323). The rate of successful genotypes was above 95% for each SNP.

### Association testing

All frequent SNPs (MAF>5%) identified in the *VEGF* gene were tested for association with the log-transformed VEGF adjusted for age phenotype in the detection samples. The genotype frequencies of the tested SNPs are reported in a supplementary table (Table S3). Significant associations were then confirmed in the population sample of each village (656 individuals in Campora, 852 in Gioi and 449 in Cardile). To test for association while taking into account the relatedness between individuals, the phenotypes were regressed on the genotypes and a Wald Test was applied on the least square estimator of β (regression coefficient for the genotype covariate in the regression) with a variance of the estimator modified to account for the relatedness, using the genealogical information [9].

To correct for multiple testing, we applied the procedure proposed by Nyholt [32] and modified by Li and Ji [33]. Briefly, a number of independent tests (Meff) equivalent to the number of correlated SNPs tested was estimated from the LD pattern among the SNPs and a Bonferroni correction for Meff tests was applied to obtain the corrected p-value threshold.

The global comparison of LD among the rs25648, rs3025039, rs3025020 and rs41282644 SNPs in the population samples was conducted using the approach proposed by Zaykin et al [11]. Based on the composite LD coefficient proposed by Weir and Cockerham [34], this test contrasts the LD matrices with an empirical assessment of type I error. To account for the inter-individual relationship, all measures of LD were computed on sub-samples of poorly related individuals: 163 individuals for the Campora *population sample*, 104 individuals for the Cardile *population sample* and 111 individuals for the Gioi *population sample.*

The haplotypes were reconstructed taking advantage of family information and tested for association with the VEGF levels using the software FBAT. A biallelic test was performed in which each haplotype was tested against all the others pooled together and an additive model was applied. Two analyses were carried out successively. One used the haplotypes made of the three SNPs associated in Campora and the other used the haplotypes made of the four associated SNPs (three associated in Campora and one associated in Cardile). Only haplotypes having a frequency >1% were tested for association with the VEGF levels.

## Supporting Information

Figure S1 A) Schematic representation of the *VEGF* gene. The exons are reported in black, introns in white, regulative regions in dark grey, and promoter region in light grey. The position of the 56 SNPs identified in the gene is also indicated. The four SNPs associated with the VEGF levels are framed. B) LD patterns between the four associated SNPs in Campora, Gioi and Cardile. R-squared values are indicated.
(TIF)

Table S1 Characteristics of the sub-pedigree sets used in the linkage study for the VEGF serum levels in Campora, Gioi and Cardile
(DOC)

Table S2 List of the primers designed to sequence *VEGF* gene
(DOC)

Table S3 Genotype frequencies in the *detection samples* of the 26 SNPs analyzed for association with the VEGF levels.
(DOC)
