# Supplementary figures and images for: Genetics of VEGF Serum Variation in Human Isolated Populations of Cilento: Importance of VEGF Polymorphisms
Source: PLoS One. 2011 Feb 9;6(2):e16982. doi: 10.1371/journal.pone.0016982 (PMC3036731; doi:10.1371/journal.pone.0016982)

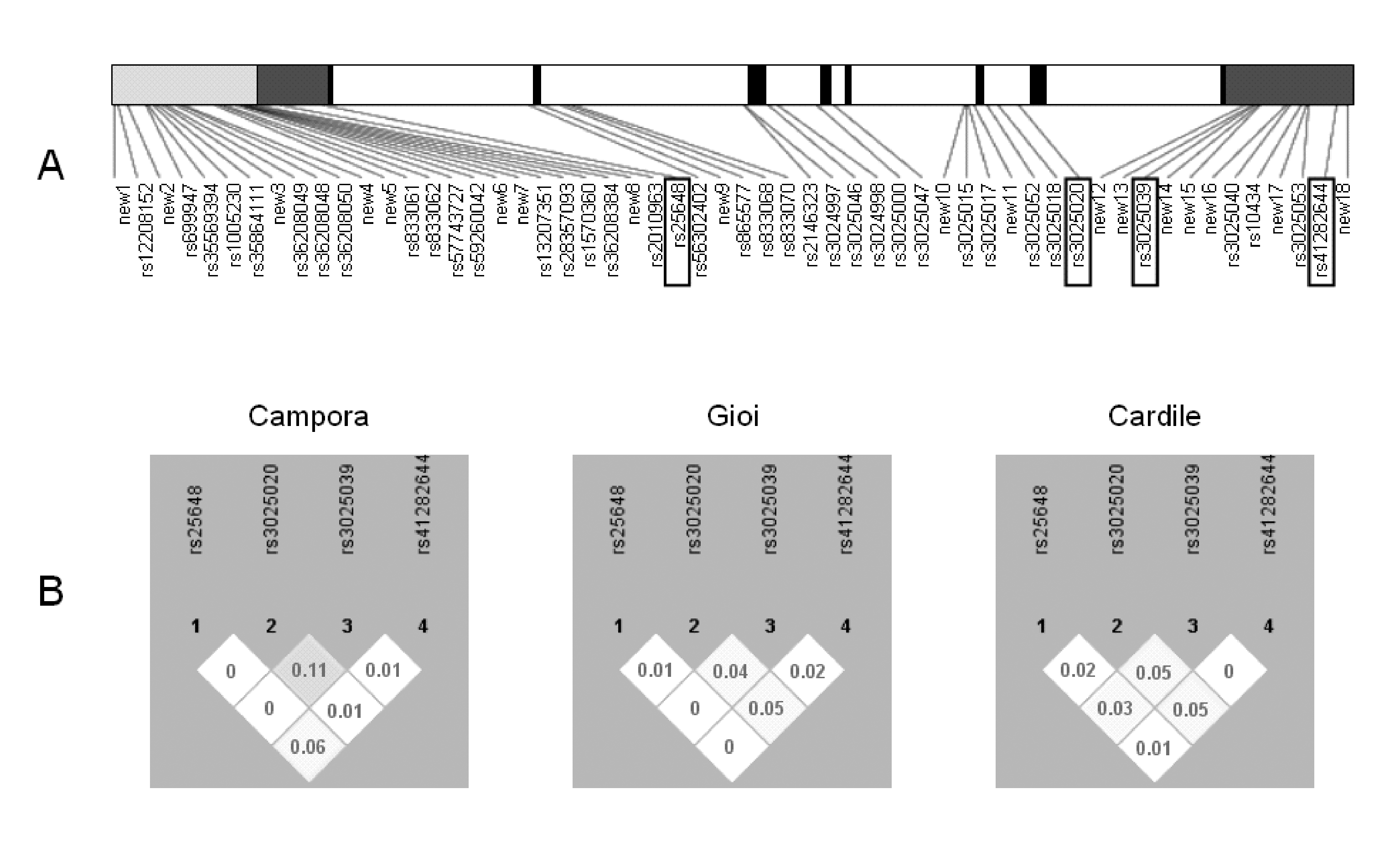

Supplement: Figure S1 — A) Schematic representation of the VEGF gene. The exons are reported in black, introns in white, regulative regions in dark grey, and promoter region in light grey. The position of the 56 SNPs identified in the gene is also indicated. The four SNPs associated with the VEGF levels are framed. B) LD patterns between the four associated SNPs in Campora, Gioi and Cardile. R-squared values are indicated. (TIF) [file pone.0016982.s001.tif]
