# Supplementary material for: Genetics of VEGF Serum Variation in Human Isolated Populations of Cilento: Importance of VEGF Polymorphisms
Source: PLoS One. 2011 Feb 9;6(2):e16982. doi: 10.1371/journal.pone.0016982 (PMC3036731; doi:10.1371/journal.pone.0016982)
Supplement: Table S1 — Characteristics of the sub-pedigree sets used in the linkage study for the VEGF serum levels in Campora, Gioi and Cardile (DOC) [file pone.0016982.s002.doc]

Table S1. Characteristics of the sub-pedigree sets used in the linkage study for the VEGF serum levels in Campora, Gioi and Cardile

| **Village** | **N° of sub-pedigree sets** | **Mean number of families/sub-pedigree set** | **Mean number of individuals/ family** | **Mean number of phenotyped individuals/ family** | **Mean number of generations/family** | **Kinship between individuals/family**  **(mean ± SD)** |
| --- | --- | --- | --- | --- | --- | --- |
| **Campora** | 15 | 96.3 | 10.7 | 5.9 | 3.6 | 0.15 ± 0.08 |
| **Gioi** | 16 | 137.3 | 9.0 | 5.2 | 3.4 | 0.16 ± 0.08 |
| **Cardile** | 18 | 72.6 | 9.6 | 5.4 | 3.5 | 0.16 ± 0.08 |
