# Supplementary material for: Genetics of VEGF Serum Variation in Human Isolated Populations of Cilento: Importance of VEGF Polymorphisms
Source: PLoS One. 2011 Feb 9;6(2):e16982. doi: 10.1371/journal.pone.0016982 (PMC3036731; doi:10.1371/journal.pone.0016982)
Supplement: Table S2 — List of the primers designed to sequence VEGF gene (DOC) [file pone.0016982.s003.doc]

**Table S2**: List of the primers designed to sequence *VEGF* gene

| **GENE REGION** | **PRIMER SEQUENCE** | |
| --- | --- | --- |
| Promoter-1 | Forward | 5' - CCAGGTCACAGCCAGGTTAT - 3' |
|  | Reverse | 5' - GTTCCATCGGTATGGTGTCC - 3' |
|  |  |  |
| Promoter-2 | Forward | 5' - CCAAGTGGTGGAGACAGGAC - 3' |
|  | Reverse | 5' - GCCAGCACTAAGGAACGTCT - 3' |
|  |  |  |
| Promoter-3 | Forward | 5' - GAGACGAAACCCCCATTTCT - 3' |
|  | Reverse | 5' - CACACGTCCTCACTCTCGAA - 3' |
|  | | |
| Promoter-4 | Forward | 5' - AGCTGTTTGGGAGGTCAGAA - 3' |
|  | Reverse | 5' - CTGACCGGTCCACCTAACC - 3' |
|  | | |
| 5'UTR | Forward | 5' - TTTTTAAAAGTCGGCTGGTAGC - 3' |
|  | Reverse | 5' - AGAACAGCCCAGAAGTTGGA - 3' |
|  |  |  |
| Exon1 | Forward | 5' - CCCCAGCTACCACCTCCT - 3' |
|  | Reverse | 5' - CCCTGCACCTAAGACGACAG - 3' |
|  |  |  |
| Exon2 | Forward | 5' - TCGACAGTGAAGCATTCTGG - 3' |
|  | Reverse | 5' - CTCACACAGGAAGGGTCACA - 3' |
|  |  |  |
| Exon3 | Forward | 5' - TGGAATGAAAACAGGCCTTC - 3' |
|  | Reverse | 5' - GGCACTCAGGACTCTCTCCA - 3' |
|  |  |  |
| Exon4-5 | Forward | 5' - CCTGAGCCTCTTTCCTGCTA - 3' |
|  | Reverse | 5' - AGTCCCCCACAAGACCAAGT - 3' |
|  |  |  |
| Exon6 | Forward | 5' - GACTGGGAGCCACTGTGAGT - 3' |
|  | Reverse | 5' - GCAGAAGCCTAGAGCAATGG - 3' |
|  |  |  |
| Exon7 | Forward | 5' - GACACAGCATTGCCCCTTAT - 3' |
|  | Reverse | 5' - AATCGGCTTTCAGCTTCTCA - 3' |
|  |  |  |
| Exon8 | Forward | 5' - CACCTTCCTGTCCTCTCTGC - 3' |
|  | Reverse | 5' - TGTTCCCAAAACTGGGTCAT - 3' |
|  |  |  |
| 3'UTR-1 | Forward | 5' - TGACAGGGAAGAGGAGGAGA - 3' |
|  | Reverse | 5' - CCGGTACAAATAAGAGAGCAAGA - 3' |
|  |  |  |
| 3'UTR-2 | Forward | 5' - TGTGGAGGCAGAGAAAAGAGA - 3' |
|  | Reverse | 5' - CGGGAGGAAGTCTAGAGCAA - 3' |
