# Supplementary material for: Genetics of VEGF Serum Variation in Human Isolated Populations of Cilento: Importance of VEGF Polymorphisms
Source: PLoS One. 2011 Feb 9;6(2):e16982. doi: 10.1371/journal.pone.0016982 (PMC3036731; doi:10.1371/journal.pone.0016982)
Supplement: Table S3 — Genotype frequencies in the detection samples of the 26 SNPs analyzed for association with the VEGF levels. (DOC) [file pone.0016982.s004.doc]

**Table S3.** Genotype frequencies in the *detection samples* of the 26 SNPs analyzed for association with the VEGF levels*.*

| **Polymorphism** | **gene location** | **type** | **Genotype frequencies** | | |
| --- | --- | --- | --- | --- | --- |
|  |  |  | **Campora (N=42)** | **Gioi (N=49)** | **Cardile (N=45)** |
| rs699947 | Promoter | A/C | A/A=0.12 A/C=0.57 C/C=0.31 | A/A=0.17 A/C=0.52 C/C=0.31 | A/A=0.22 A/C=0.43 C/C=0.35 |
| rs35569394 | Promoter | Ins/Del 18 bp | Ins/Ins=0.12 Ins/Del=0.57 Del/Del=0.31 | Ins/Ins=0.18 Ins/Del=0.52 Del/Del=0.30 | Ins/Ins=0.22 Ins/Del=0.43 Del/Del=0.35 |
| rs1005230 | Promoter | C/T | T/T=0.12 C/T=0.57 C/C=0.31 | T/T=0.18 C/T=0.52 C/C=0.30 | T/T=0.22 C/T=0.43 C/C=0.35 |
| rs35864111 | Promoter | –/G | –/–=0.12  –/G=0.57 G/G=0.31 | –/–=0.18  –/G=0.52 G/G=0.30 | –/–=0.18  –/G=0.53 G/G=0.29 |
| rs36208049 | Promoter | G/T | T/T=0.00 G/T=0.12 G/G=0.88 | T/T=0.02 G/T=0.06 G/G=0.92 | T/T=0.00 G/T=0.18 G/G=0.82 |
| rs833061 | Promoter | C/T | T/T=0.31 C/T=0.21 C/C=0.48 | T/T=0.24 C/T=0.52 C/C=0.24 | T/T=0.23 C/T=0.53 C/C=0.24 |
| rs13207351 | Promoter | A/G | G/G=0.31 A/G=0.21 A/A=0.48 | G/G=0.24 A/G=0.50 A/A=0.24 | G/G=0.23 A/G=0.53 A/A=0.24 |
| rs1570360 | Promoter | A/G | G/G=0.26 A/G=0.40 A/A=0.34 | G/G=0.40 A/G=0.42 A/A=0.18 | G/G=0.43 A/G=0.50 A/A=0.07 |
| rs2010963 | 5'UTR | C/G | C/C=0.17 C/G=0.52 G/G=0.31 | C/C=0.12 C/G=0.46 G/G=0.42 | C/C=0.14 C/G=0.52 G/G=0.34 |
| rs25648 | 5'UTR | C/T | T/T=0.00 C/T=0.17 C/C=0.83 | T/T=0.02 C/T=0.12 C/C=0.86 | T/T=0.02 C/T=0.35 C/C=0.63 |
| rs865577 | intron 2 | G/T/C | C/C=0.07 C/G=0.24 G/G=0.69 | C/C=0.09 C/G=0.43 G/G=0.48 | C/C=0.18 C/G=0.40 G/G=0.42 |
| rs833068 | intron 2 | A/G | A/A=0.17 A/G=0.54 G/G=0.29 | A/A=0.11 A/G=0.48 G/G=0.41 | A/A=0.17 A/G=0.54 G/G=0.29 |
| rs833070 | intron 2 | C/T | T/T=0.12 C/T=0.57 C/C=0.31 | T/T=0.14 C/T=0.53 C/C=0.33 | T/T=0.18 C/T=0.53 C/C=0.29 |
| rs2146323 | intron 2 | A/C | A/A=0.07 A/C=0.52 C/C=0.41 | A/A=0.04 A/C=0.31 C/C=0.65 | A/A=0.00 A/C=0.56 C/C=0.44 |
| rs3024997 | intron 2 | A/G | A/A=0.17 A/G=0.48 G/G=0.35 | A/A=0.08 A/G=0.47 G/G=0.45 | A/A=0.13 A/G=0.56 G/G=0.31 |
| rs3024998 | intron 3 | C/T | T/T=0.17 C/T=0.50 C/C=0.33 | T/T=0.10 C/T=0.47 C/C=0.43 | T/T=0.16 C/T=0.53 C/C=0.31 |
| rs3025000 | intron 3 | C/T | T/T=0.12 C/T=0.40 C/C=0.48 | T/T=0.04 C/T=0.45 C/C=0.51 | T/T=0.07 C/T=0.58 C/C=0.35 |
| rs3025017 | intron 5 | A/G | A/A=0.00 A/G=0.24 G/G=0.76 | A/A=0.00 A/G=0.16 G/G=0.84 | A/A=0.00 A/G=0.17 G/G=0.83 |
| rs3025052 | intron 6 | C/T | T/T=0.00 C/T=0.02 C/C=0.98 | T/T=0.00 C/T=0.04 C/C=0.96 | T/T=0.00 C/T=0.14 C/C=0.86 |
| rs3025018 | intron 6 | C/G/T | G/T=0.05 C/G=0.12 C/T=0.10 C/C=0.73 T/T=0.00 G/G=0.00 | G/T=0.00 C/G=0.06 C/T=0.18 C/C=0.74 T/T=0.00 G/G=0.02 | G/T=0.00 C/G=0.05 C/T=0.21 C/C=0.74 T/T=0.00 G/G=0.00 |
| rs3025020 | intron 6 | C/T | C/C=0.34 C/T=0.40 T/T=0.26 | C/C=0.55 C/T=0.37 T/T=0.08 | C/C=0.61 C/T=0.34 T/T=0.05 |
| rs3025039 | 3'UTR | C/T | T/T=0.00 C/T=0.29 C/C=0.71 | T/T=0.02 C/T=0.20 C/C=0.78 | T/T=0.00 C/T=0.36 C/C=0.64 |
| rs3025040 | 3'UTR | C/T | T/T=0.00 C/T=0.26 C/C=0.74 | T/T=0.02 C/T=0.19 C/C=0.79 | T/T=0.00 C/T=0.36 C/C=0.64 |
| rs10434 | 3'UTR | A/G | A/A=0.07 A/G=0.33 G/G=0.60 | A/A=0.18 A/G=0.39 G/G=0.43 | A/A=0.27 A/G=0.49 G/G=0.24 |
| rs3025053 | 3'UTR | A/G | A/A=0.00 A/G=0.14 G/G=0.86 | A/A=0.00 A/G=0.12 G/G=0.88 | A/A=0.00 A/G=0.16 G/G=0.84 |
| rs41282644 | 3'UTR | A/G | A/A=0.00 A/G=0.00 G/G=1.00 | A/A=0.00 A/G=0.14 G/G=0.86 | A/A=0.00 A/G=0.20 G/G=0.80 |
